# Epoxyqueuosine Reductase Structure Suggests a Mechanism for Cobalamin-dependent tRNA Modification*

**DOI:** 10.1074/jbc.M115.685693

**Published:** 2015-09-16

**Authors:** Karl A. P. Payne, Karl Fisher, Hanno Sjuts, Mark S. Dunstan, Bruno Bellina, Linus Johannissen, Perdita Barran, Sam Hay, Stephen E. J. Rigby, David Leys

**Affiliations:** From the Manchester Institute of Biotechnology, University of Manchester, Princess Street 131, Manchester M1 7DN, United Kingdom

**Keywords:** crystal structure, electron paramagnetic resonance (EPR), enzyme mechanism, nucleic acid enzymology, RNA modification, transfer RNA (tRNA), iron sulfur protein, queuosine biosynthesis, vitamin B12

## Abstract

Queuosine (Q) is a hypermodified RNA base that replaces guanine in the wobble positions of 5′-GUN-3′ tRNA molecules. Q is exclusively made by bacteria, and the corresponding queuine base is a micronutrient salvaged by eukaryotic species. The final step in Q biosynthesis is the reduction of the epoxide precursor, epoxyqueuosine, to yield the Q cyclopentene ring. The epoxyqueuosine reductase responsible, QueG, shares distant homology with the cobalamin-dependent reductive dehalogenase (RdhA), however the role played by cobalamin in QueG catalysis has remained elusive. We report the solution and structural characterization of *Streptococcus thermophilus* QueG, revealing the enzyme harbors a redox chain consisting of two [4Fe-4S] clusters and a cob(II)alamin in the base-off form, similar to RdhAs. In contrast to the shared redox chain architecture, the QueG active site shares little homology with RdhA, with the notable exception of a conserved Tyr that is proposed to function as a proton donor during reductive dehalogenation. Docking of an epoxyqueuosine substrate suggests the QueG active site places the substrate cyclopentane moiety in close proximity of the cobalt. Both the Tyr and a conserved Asp are implicated as proton donors to the epoxide leaving group. This suggests that, in contrast to the unusual carbon-halogen bond chemistry catalyzed by RdhAs, QueG acts via Co-C bond formation. Our study establishes the common features of Class III cobalamin-dependent enzymes, and reveals an unexpected diversity in the reductive chemistry catalyzed by these enzymes.

## Teaser

**Background:** Little is known about epoxyqueuosine reductase (QueG), which catalyzes the final step in the biosynthesis of queuosine.

**Results:** We report solution and structural characterization of *Streptococcus thermophilus* QueG.

**Conclusion:** The QueG similarity to reductive dehalogenases is largely limited to cofactor binding.

**Significance:** Our study establishes the link between cobalamin-metabolism and tRNA modification and suggests a mechanism for cobalamin-dependent epoxide reduction.

## Introduction

**FIGURE 1.**
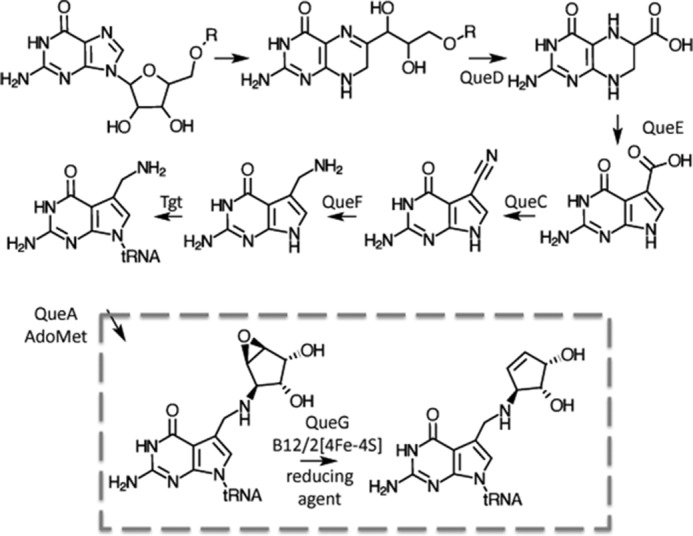
**Bacterial queuosine biosynthetic pathway**.

**FIGURE 2.**
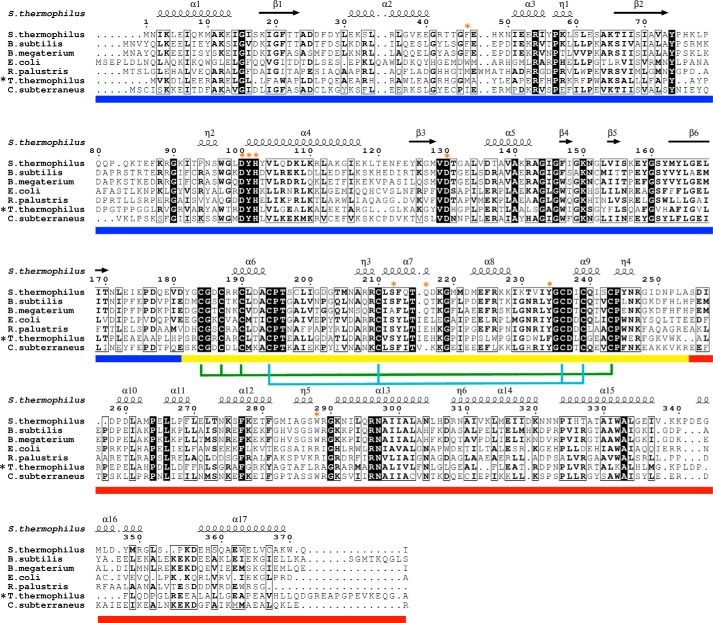
**Multiple sequence alignment of QueG homologues from *S. thermophilus* (WP̱011227267), *B. subtilis* (CAB07527), *B. megaterium* (YP̱003595681), *E. coli* (NP̱418587), *Rhodopseudomonas palustris* (WP̱011661955), *Thermus thermophilus* (WP̱011172569), and *Caldanaerobacter subterraneus* (WP̱011025966).** Secondary structure elements of the *S. thermophilus* QueG crystal structure are shown. α-helices and 3_10_ helices (denoted as η) are shown as *squiggles* and β-strands as *arrows*. The colored bar denotes the domain structure using the same color scheme as Fig. 6, with the N-terminal RdhA-like domain in *blue*, the ferredoxin domain in *yellow* and the C-terminal domain in *red*. Cysteines involved in coordinating iron-sulfur cluster 1 (adjacent to the cobalamin) are denoted by the *cyan lines*, those that coordinate cluster 2 by *green lines* and active site residues highlighted in Fig. 11 are indicated by *orange stars*. In early studies, queuosine could not be detected in the tRNAs of *Thermus thermophilus* (*black star*) (34).

Transfer RNAs (tRNA)^4^ undergo a wide variety of post-transcriptional nucleotide modifications that contribute to tRNA stability, tRNA recognition, translational accuracy, and the decoding of degenerate codons. These modifications range from simple methylation through to the formation of complex hypermodified bases. Biosynthesis of hypermodified bases, such as wyebutoxine, archaeosine, and queuosine, involves multiple enzymatic steps. The latter two share a common 7-deazaguanosine core but differ in the extent of further decoration. Whereas archaeosine is found in the dihydrouridine loop of archaeal tRNAs, queuosine is found in position 34 (the “wobble” position) of the anti-codon of 5′-GUN-3′ tRNAs (encoding tyrosine, histidine, asparagine, and aspartate) and is essentially ubiquitous among bacteria and eukaryotes (1). Q biosynthesis is exclusively performed in bacteria, and the corresponding queuine base is a micronutrient salvaged by eukaryotic species (recently reviewed by Fergus *et al.* (2)). In bacteria, the biosynthesis of Q consists of 8 steps (Fig. 1), beginning with the conversion of guanosine triphosphate (GTP) to 7,8-dihydroneopterin triphosphate (H_2_NTP) by GTP cyclohydrolase I. QueD converts H_2_NTP to 6-carboxy-5,6,7,8-tetrahydropterin, which then undergoes ring contraction catalyzed by QueE to produce 7-carboxy-7-deazaguanine. QueC converts the carboxyl moiety to a nitril group, which in turn is reduced by QueF to produce 7-aminomethyl-deazaguanine, otherwise known as preQ_1_ (3–5). Following tRNA guanine transglycosylase (Tgt) insertion of preQ_1_ into the tRNA (6), the final two steps in the Q biosynthesis occur on the tRNA substrate. QueA catalyzes the transfer and isomerization of an *S*-adenosylmethionine-derived ribose to the tRNA 7-aminomethyl-deazaguanosine to form epoxyqueuosine-tRNA (oQ) (7). The final step in Q biosynthesis involves the reduction of the oQ epoxide moiety to form the Q cyclopentenediol group. Until recently, the identity of the enzyme responsible remained elusive, although it was demonstrated the final step was dependent on cobalamin (8). Screening of *Escherichia coli* single gene knock-out mutants that accumulated oQ led to the discovery of *yjeS,* a gene of unknown function that was identified as the structural gene for the oQ reductase and thus termed QueG (9). QueG shares distant sequence homology with the reductive dehalogenases, a class of iron-sulfur/cobalamin-dependent enzymes that catalyze the key step in organohalide respiration (10). The latter currently constitute the sole members of the third subfamily of cobalamin-dependent enzymes, the other subfamilies being the adenosylcobalamin-dependent isomerases and the methylcobalamin-dependent methyltransferases, respectively (11). In contrast to the latter, the subfamily three members bind Cob(II)alamin in a pentacoordinate, base-off form. Spectroscopic characterization of the *Bacillus subtilis* QueG confirmed it also displaces the dimethylbenzimidazole of cobalamin upon binding to the protein (12). Despite these recent insights, little is known about the QueG mechanism due to a lack of detailed structural insight. Although the structures of two reductive dehalogenases have been recently reported (13, 14), clear sequence homology between QueG and the reductive dehalogenases is largely limited to the ferredoxin domain, while the QueG-specific DYH motif implicated in catalysis (Fig. 2) is not present in the reductive dehalogenases. To provide detailed insight into oQ reduction and explore the common structural features of the third subfamily of cobalamin-dependent enzymes, we determined the crystal structure of the *Streptococcus thermophilus* QueG. Combined with detailed spectroscopic characterization and substrate docking, our data reveal the similarity with the reductive dehalogenases is limited to the relative position of the redox cofactors and the presence of a single conserved Tyr in the active site. The distinct protein environments surrounding the conserved tyrosine, combined with the widely differing chemical nature of the transformations catalyzed, suggest a potential for substantial mechanistic diversity in this subfamily of the cobalamin and iron-sulfur-dependent oxidoreductases.

## Experimental Procedures

### 

#### 

##### Cloning of S. thermophilus QueG for B. megaterium Heterologous Expression

The *S. thermophilus QueG* gene was PCR amplified using primers StpNHis1622F (CAAGGCGCCCAGATCTCAATGAATATCAAACTGGAAATCCAGAAAATG) and StpNHis1622R (GGCCGGTACCGGATCCTTAAATCTGCCATTTCGCACAAACG) using Phusion polymerase (NEB). The PCR product was cloned into the BglII and BamHI sites of pN-His-TEV1622 plasmid (15) using Infusion HD (Clontech) and transformed into *E. coli* NEB5α. Once the sequence of the insert was confirmed the purified plasmid was transformed into *B. megaterium* DSM319, using the modified minimal medium protoplast transformation protocol (16).

##### Heterologous Expression of S. thermophilus QueG in B. megaterium

*B. megaterium* transformants were grown in terrific broth supplemented with 10 μg/ml tetracycline at 37 °C/180 rpm until the culture reached an *A*_578_ ∼0.4. Cultures were supplemented with 50 μm ammonium iron(II) sulfate, 1 μm B12 and induced with 0.1% xylose. Cultures were grown overnight at 17 °C/180 rpm and then harvested by centrifugation (4 °C, 7000 × *g* for 10 min).

##### Purification of S. thermophilus QueG

Cell pellets were resuspended in buffer A (400 mm NaCl, 100 mm NaP_i_, pH 7.5) supplemented with DNase, lysozyme (Sigma), and a Complete EDTA-free protease inhibitor mixture (Roche). Cells were lysed using a French press at 20000 psi and the lysate clarified by centrifugation at 125,000 × *g* for 90 min. The supernatant was applied to a Ni-NTA-agarose column (Qiagen). The column washed successively with 3 column volumes of buffer A supplemented with 10 mm imidazole and protein eluted in 1-ml fractions with buffer A supplemented with 250 mm imidazole. Imidazole was removed using a 10-DG desalting column (Bio-Rad) equilibrated with Heparin Binding buffer (100 mm NaCl, 25 mm Tris, pH 7.5). Protein was applied to a 5-ml heparin column (GE Healthcare) and the column washed with 2 column volumes of heparin binding buffer, protein was eluted with a gradient of 100–500 mm NaCl over 20 column volumes. Samples were subjected to SDS-PAGE analysis and fractions found to contain the purified protein were pooled. While initial QueG preparations were performed aerobically, subsequent preparations were performed anaerobically using N_2_ purged buffers and a 100% N_2_-atmosphere glove box (Belle Technology, UK).

##### Size Exclusion Chromatography Coupled to Multi-Angle Light Scattering (SEC-MALS)

Size exclusion chromatography coupled with multi-angle light scattering (SEC-MALS) analysis was performed at 25 °C. 500 μl of 1.5 mg/ml protein was loaded onto a Superdex 200 10/300GL column (GE Life-Sciences, 0.75 ml/min in 100 mm NaCl, 25 mm Tris/Cl, pH 7.5) and passed through a Wyatt DAWN Heleos II EOS 18-angle laser photometer coupled to a Wyatt O ptilab rEX refractive index detector. Data were analyzed using Astra 6 software (Wyatt Technology Corp.).

##### Native Protein Mass Spectrometry

Protein was buffer exchanged into anaerobic 100 mm ammonium acetate, pH 6.8 using a 10-DG column (Bio-Rad) and any precipitated protein removed by centrifugation. MS data were acquired on a Waters Synapt G2 mass spectrometer operating in ToF mode. The protein complex was infused using a nano-ESI source. The spray voltage was optimized for signal at 1.3 kV, and the source temperature was set at 80 °C. Sampling and extraction cone were set at 20 V and 3 V, respectively.

##### UV-Vis Spectroscopy/Protein Quantification

UV-Vis absorbance spectra were recorded with a Cary UV-Vis spectrophotometer. B12 was extracted from QueG by incubating the protein with 1 mm KCN at 100 °C for 5 min followed by centrifugation to remove precipitated protein. To obtain the reduced *S. thermophilus* QueG spectrum, 134 μm enzyme was incubated anaerobically with 25 μm 5-deazariboflavin and 2 mm EDTA under a blue LED lamp for 1 h. Protein concentration was estimated using ϵ_280_ = 48360 m^−1^ cm^−1^ (calculated from the primary amino acid sequence using the ProtParam program on the ExPASy proteomics server).

##### EPR Spectroscopy

X-band continuous wave electron paramagnetic resonance (EPR) spectra were obtained using a Bruker ELEXSYS E500/580 spectrometer equipped with an Oxford Instruments ESR900 liquid helium cryostat and associated ITC503 temperature controller. Experimental parameters were as given in the figure caption.

##### Crystallization

Purified QueG in 100 mm NaCl, 25 mm Tris, pH 7.5, was concentrated in a Vivaspin 30 kDa cut off spin concentrator to a final concentration of 11 mg/ml. Initial screening by sitting drop was performed aerobically, mixing 0.3 μl of protein with 0.3 μl of mother liquor led to crystals in a variety of conditions when incubated at 21 °C. Anerobic crystals were obtained by mixing 2 μl of protein with 2 μl of 0.3 m sodium acetate, 0.1 m Tris/Cl, pH 7.5, 15% *w*/*v* PEG 4000 and incubated at room temperature in a 100% N_2_-atmosphere glove box (Belle Technology).

##### Diffraction Data Collection and Structure Elucidation

Crystals were flash-cooled in liquid nitrogen by supplementing the mother liquor with 10% PEG 200. Data were collected at Diamond beamlines and subsequently handled using the CCP4 suite (17). All data were reduced and scaled using XDS (18) and initial phases obtained by merging various native datasets using BLEND (19). This generated a highly redundant low-resolution data set with sufficient anomalous signal from the native Fe and Co ions to allow substructure determination and initial phasing using MLPHARE (17). Interpretable maps were obtained following density modification and 5-fold NCS averaging combined with phases extension to 2.7 Å using DM (17). An initial model was automatically generated using Buccaneer (20), and iteratively rebuilt and refined using Coot and REFMAC5 (21). Local NCS restraints were used throughout the refinement. The final model was refined using data extending to 2.65 Å and contains 5 molecules in the asymmetric unit. For final data and refinement statistics, see Table 1.

**TABLE 1:** **Data collection and refinement statistics**

	QueG (PDB code 5D6S)
**Data collection**	
Wavelength, Å	0.9
Space group	P3_1_21
Cell dimensions	*a* = 106.12, *b* = 106.12, and *c* = 332.28 Å; αandβ=90 and γ=120°
Molecules per asymmetric unit	5
Solvent content, %	49
*R*_merge_ (%)	12.6 (188.7)
*CC1/2*	0.999 (0.678)
*I*/σ*I*	17.6 (2.57)
Completeness (%)	99.8 (97.8)
Redundancy	14.0 (14.2)
Wilson B factor (Å^2^)	65.9

**Refinement**	
Resolution (Å)	88.58–2.65 (2.72–2.65)
No. reflections	60261 (4332)
*R*_work_/*R*_free_	21.95/25.73 (32.8/39.7)
No. non-hydrogen atoms	15145
Mean B factor (Å^2^)	76.53

**R.m.s. deviations**	
Bond lengths (Å)	0.015
Bond angles (°)	2.057

**Ramachandran plot**	
Favorable regions (%)	92.6
Allowed regions (%)	5.8
Outliers (%)	1.6

##### Docking

Computational docking was performed with Autodock vina (22), using AutoDock Tools 1.5.6 to assign hydrogens and Gasteiger charges, as described in Ref. 13. For simplicity epoxyqueuosine was docked, *i.e.* the attached phosphate group was truncated. However, the docked conformations chosen are compatible with an attached 5′-phosphate pointing out of the active site. The epoxyqueuosine model was built and geometry optimized in the gas phase using the UFF force field. As there are multiple stable conformations of epoxyqueuosine, all rotatable bonds were allowed to move during docking. A total of 5 active site residues were flexible during docking, namely Phe-46, Tyr-101, His-102, Asp-130, Gln-216. Docking was performed with both the doubly (positively charge) and singly protonated (neutral) epoxyqueuosine N1, with very similar results. For singly protonated N1, docking was performed with the proton in each of the two possible orientations. In each case the conformation chosen was that with the shortest distance between Co and epoxide carbons, with energies above the lowest energy conformation of only 0.2 kcal mol^−1^ for positively charged N1 and 0.0 and 0.8 kcal mol^−1^ for neutral N1.

## Results

### 

#### 

##### Heterologous Production and Solution Characterization of S. thermophilus QueG

To provide detailed structural insights for QueG, several thermophilic *queG* genes were screened for heterologous expression (data not shown), with the *S. thermophilus* QueG proving the most promising candidate. All attempts to express *S. thermophilus* QueG in *E. coli* resulted in the recombinant protein forming inclusion bodies, similar to that reported previously for expression of reductive dehalogenases in *E. coli* (23). We used *Bacillus megaterium* as an alternative expression host, which had previously proven successful for the production of a reductive dehalogenase (13). Soluble recombinant *S. thermophilus* QueG was successfully expressed in *B. megaterium* with an N-terminal poly-His-tag and purified anaerobically by Ni-affinity chromatography followed by heparin-affinity chromatography.

**FIGURE 3.**
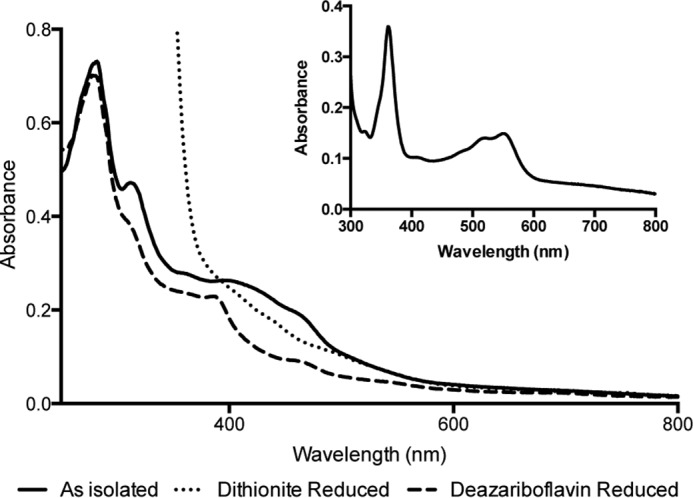
**UV-Vis spectrum of *S. thermophilus* QueG.** Spectra are shown of the protein as purified under anaerobic conditions (*solid line*), following reduction by 10 mm sodium dithionite (*dotted line*) and following reduction using deazariboflavin and EDTA (*dashed line*). The *inset* shows the spectrum of cobalamin extracted from StoQ by thermodenaturation in the presence of potassium cyanide followed by centrifugation to remove precipitated protein.

The purified *S. thermophilus* oQ reductase displays a dark brown color. UV-Vis spectroscopy of the purified protein reveals broad features between the 280 nm protein peak and 500 nm (Fig. 3), showing similarity to those reported previously for the reductive dehalogenases (13) and for the recently reported *B. subtilis* QueG (12). Reduction of the protein with 10 mm sodium dithionite resulted in a lowering of the absorption between 400 and 500 nm suggesting reduction of the iron-sulfur clusters; however, features below 400 nm were obscured by the presence of excess sodium dithionite (Fig. 3). Subsequent reduction of the protein using deazariboflavin, EDTA, and light revealed a distinct feature at 390 nm, likely arising from formation of a cob(I)alamin species. Precipitation of the protein in the presence of potassium cyanide resulted in the release of a pink compound with a UV-Vis spectrum identical to cyanocobalamin (Fig. 3). Quantification of the cob(I)alamin signal suggested 0.7 ± 0.1 cobalamin per QueG monomer, while Fe quantification using bathophenanthroline indicated 6.6 ± 0.1 mol equivalent Fe or 1.65 [4Fe-4S] clusters.

**FIGURE 4.**
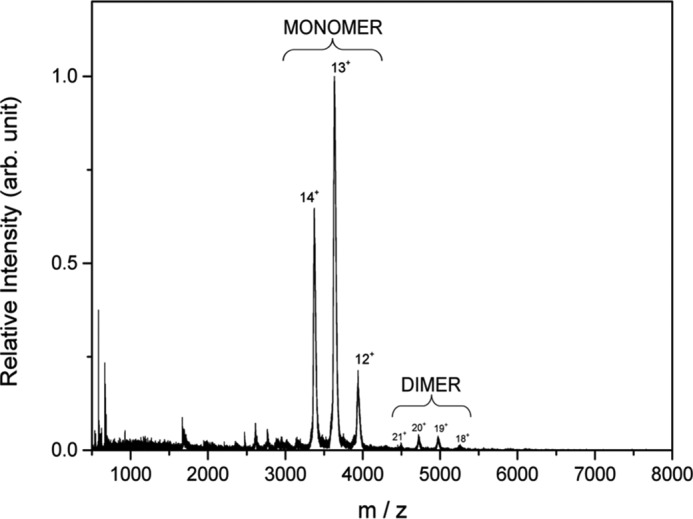
**Intact mass spectrometry of QueG.** The majority of the protein is shown to form three charge species of [M+nH]^n+^ where *n* = 12–14 giving a mass of 47,019 ± 20 Da, which is consistent with the predicted mass of the protein in complex with cobalamin and two [4Fe-4S] clusters. A small proportion of the protein (less than 5%) is shown to form a dimer as [2M+nH]^n+^ where *n* = 19 -22, and with a mass of 94,191 ± 50 Da.

To determine the oligomerization state of QueG, intact mass spectrometry was performed on protein over a range of concentrations (3–30 μm). At each concentration, the dominant species observed corresponds to the monomeric form of the protein [M+nH]^n+^ where *n* = 12–14 giving a mass of 47,019 ± 20 Da which corresponds well with the expected mass of the enzyme in complex with cobalamin and two [4Fe-4S] clusters (46,992 Da), the mass difference likely attributed to bound salt and water (Fig. 4). The narrow charge state distribution indicates that the protein that has been transferred to the gas phase is structurally homogeneic and globular (24). A small amount of dimer is also observed of the form [2M+nH]^n+^ where *n* = 19–22, and with a mass of 94191 ± 50 Da. The proportion of dimer (∼5%) is independent of protein concentration over the concentration range examined, indicating this corresponds to the ratio in solution and that the dimer is not formed due to nonspecific aggregation. To confirm that these findings apply in solution, size exclusion chromatography coupled to multi-angle light scattering (SEC-MALS) was performed. The protein eluted as a single monodisperse peak consistent with a species of ∼ 49 kDa, indicative of a monomer. No dimer could be detected by this method under the conditions used.

##### EPR Spectroscopy

**FIGURE 5.**
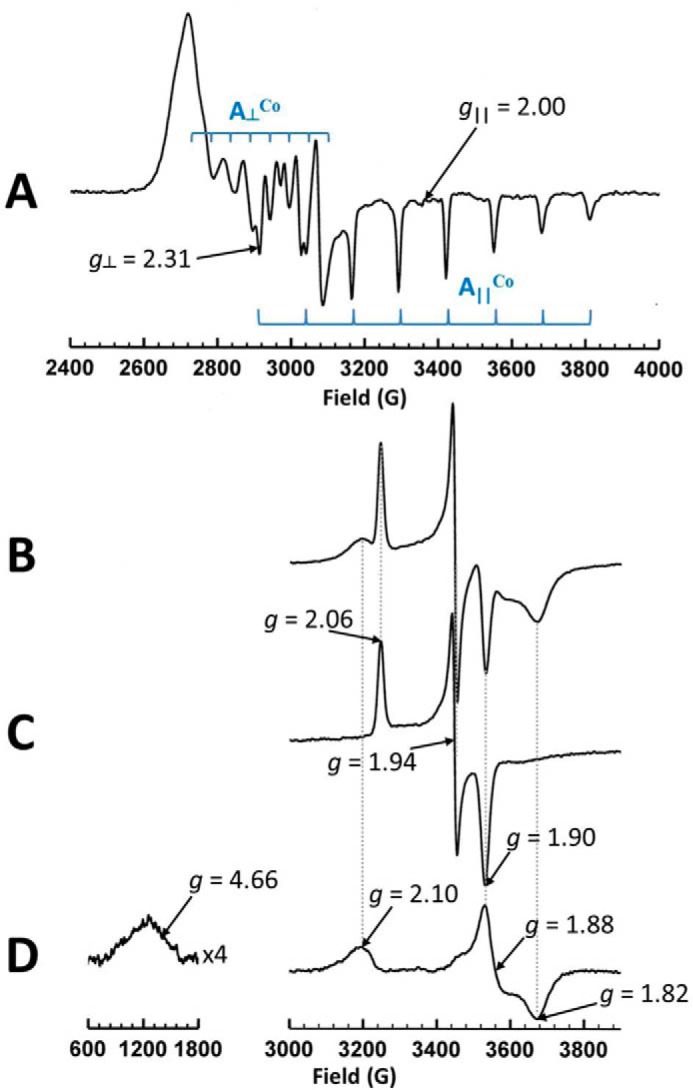
**X-band continuous wave EPR spectroscopy of *S. thermophilus* oQ reductase.** *A*, as purified at 30 K; *B*, dithionite reduced at 10 K; *C*, dithionite reduced at 20 K; and *D*, is the difference between *B* and *C* showing the second [4Fe-4S]^1+^ cluster spectrum, complete with the S = 3/2 line at low field. Cobalt hyperfine coupling is indicated in *blue* (showing the eight expected for an I = 7/2 ^59^Co). Experimental parameters: microwave power 0.5 milliwatt; modulation amplitude 4 G for *A*, 7 G for *B* and *C*; *A* is the sum of four scans; *B* and *C* are the sums of two scans each.

The X-band continuous wave EPR spectrum of QueG as purified is shown in Fig. 5*A*. Such a spectrum is characteristic of a five coordinate low spin cobalt II species having the lone unpaired electron (*i.e.* S = ½) in the d_z_2 orbital (25). Furthermore the g and A^Co^ values exhibited by this spectrum indicate that it arises from “base off” cob(II)alamin with water as the fifth ligand to the cobalt ion. Analysis of cobalt content presented above indicates that the cob(II)alamin is B12. This spectrum is very similar to that reported for B12 in RdhA (9), although significantly lacking in the chloride superhyperfine coupling in the A despite the presence of chloride ion in the buffer employed, as is the case for *B. subtilis* QueG (12). Double integration estimated this signal as 0.65 ± 0.05 spins per molecule. Reduction of this protein under anaerobic conditions using sodium dithionite yielded the spectrum of Fig. 5*B* at 10 K. This spectrum appears to derive from the spectral contributions of the S = ½ states of two [4Fe-4S]^1+^ clusters (26), with one contribution having a much smaller line width than the other. Double integration estimated these signals as totalling 1.5 ± 0.2 spins per molecule. While contributions from each of the clusters are clearly distinguishable at the extremes of the spectrum, the situation around g = 1.90 is less clear. However, Fig. 5*C* shows the spectrum of the same sample taken at 20 K in which the broad contribution is no longer visible while the narrower contribution is essentially unperturbed. This reveals a rhombic [4Fe-4S]^1+^ spectrum having g values *g*_z_ = 2.06, *g*_y_ = 1.94, and *g*_x_ = 1.90. This spectrum was then used to form the difference spectrum between Fig. 5, *B* and *C*, shown as Fig. 5*D* in which the broad contribution to Fig. 5*B* is isolated. The large line width of this spectrum suggests a significant effect of g-strain (27) that typically arises from a mobile cluster environment that is trapped into a number of conformations on freezing. This spectrum exhibits the relatively unusual g values, *g*_z_ = 2.10, *g*_y_ = 1.88, and *g*_x_ = 1.82, often associated with the S = ½ state of a [4Fe-4S]^1+^ cluster having significantly strained cysteine ligand geometry or a non-cysteine ligand (28, 29). Such clusters often exhibit EPR signals from the S = 3/2 state and the low field region of the difference spectrum Fig. 5*D* shows a weak line at *g* = 4.66 that may arise from that state.

##### QueG Crystal Structure Determination

**FIGURE 6.**
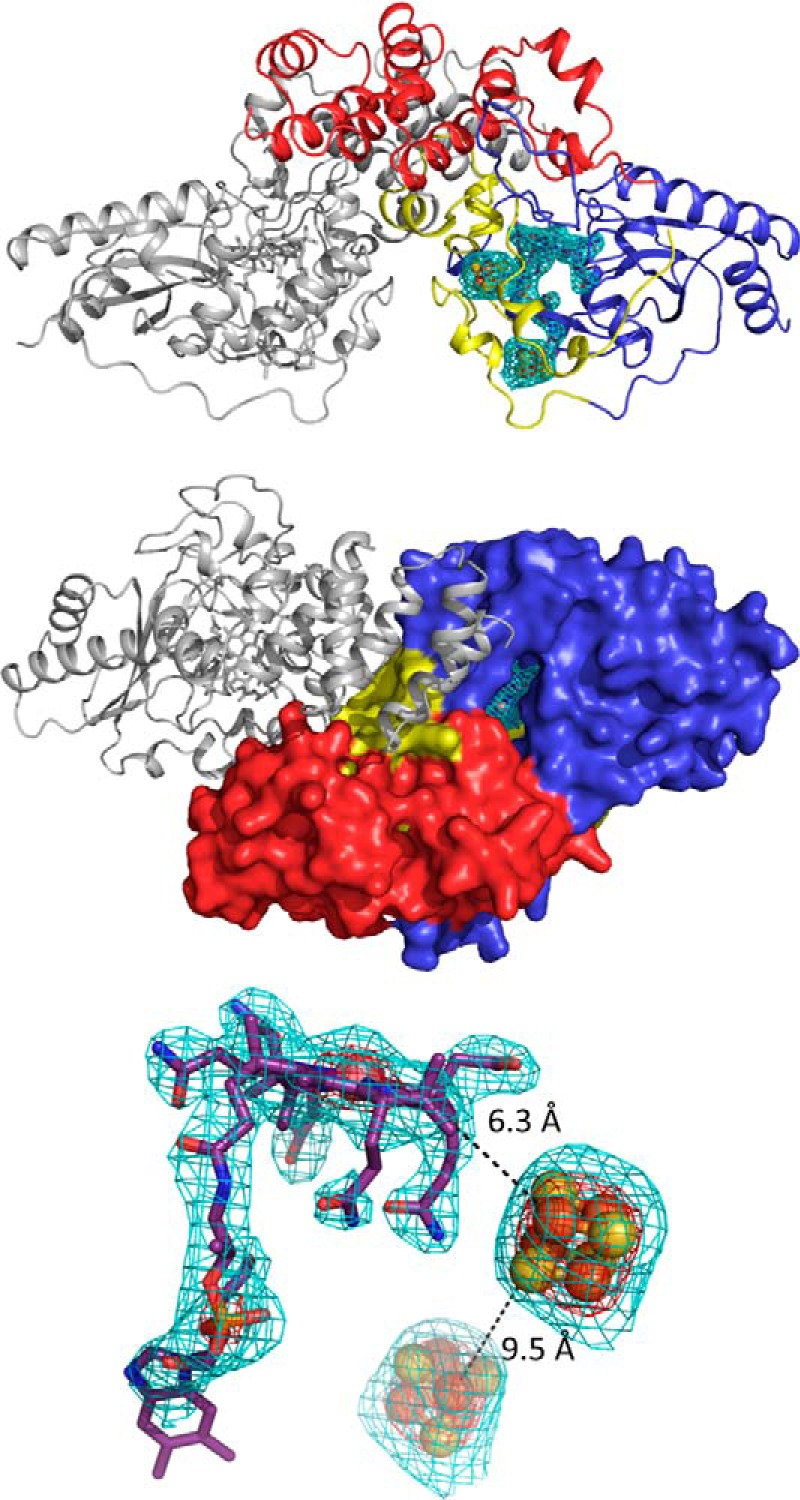
**QueG crystal structure.** *A*, schematic representation of a QueG monomer color coded as follows: N-terminal cobalamin binding region in *blue*, ferredoxin in *yellow*, and the C-terminal domain specific to QueG in *red*. The cofactors are shown in atom colored sticks with the corresponding F_o_F_c_ omit map contoured at 3 sigma shown as a *cyan* mesh. *B*, solvent accessible surface of a QueG monomer color coded as in *panel A*. The bound cobalamin resides at the bottom of a deep solvent accessible cavity. The QueG orientation is related to that in *panel A* by a 90-degree rotation along the horizontal axis. A second QueG monomer is shown in *gray* schematic depiction illustrating the putative dimeric form observed in the crystal structure. *C*, detailed view of the three cofactors bound to QueG with the corresponding F_o_F_c_ omit map contoured at 3 and 10 sigma, shown as a *cyan* and *red mesh*, respectively. *Black dotted lines* indicate the closest Fe-Fe distance and the closest Fe to corrin distance.

Initial screening of crystallization conditions was performed aerobically using aerobically prepared protein. Although a number of conditions gave rise to orange/brown crystals overnight, these crystals diffracted poorly and lost color over time, suggesting degradation of the iron-sulfur clusters. We subsequently repeated protein preparation, crystallization, and crystal harvesting under anaerobic conditions, leading to significantly improved x-ray diffraction. The anaerobic QueG crystal structure contains 5 monomers in the asymmetric unit (AU). No obvious differences can be observed between the individual molecules, with an r.m.s.d. between monomers ranging from 0.1–0.2 Å. Monomer A has been used for detailed description and comparisons with other proteins in this manuscript. Although each monomer predominantly interacts with an adjacent monomer in similar manner, suggesting a putative dimeric form of the enzyme, the associated dimer interface is relative small (1090 Å^2^ or 6% of the total accessible surface). Indeed, little evidence for dimer formation could be found using native MS and MALS analysis. This suggests the predominant species in solution corresponds to the QueG monomer (Fig. 6). The QueG monomer has an overall ovoid shape, and consists of an N-terminal cobalamin binding region (residues 1–181, a two [4Fe-4S] bacterial ferredoxin region (residues 182–257) and a C-terminal tandem α-helical repeat domain (residues 258–372). Various extended loop structures establish extensive contacts between the three regions, suggesting little scope for domain mobility. This is further supported by the similar overall structures observed for the five individual monomers in the AU. Electron density for the three redox cofactors is well defined, with the corrin plane located at the bottom of a deep surface pocket, providing direct access to the cobalt ion from the solvent (Fig. 6). The surface pocket is located at the interface between the three distinct regions. The cobalamin is bound in a pentacoordinate, base-off form by a nitroreductase-type module, similar to what is observed for the reductive dehalogenases (13, 14) and the distantly related vitamin B12 processing enzyme CblC (30).

**FIGURE 7.**
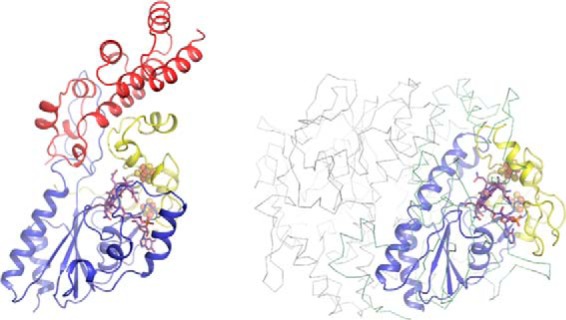
**The left structure shows QueG in an orientation that places the nitroreductase-like Rossmann fold beta sheet near perpendicular to the viewing plane.** The right structure shows the *S. multivorans* RdhA dimer, placing one monomer (in color) in a similar orientation as the QueG structure while the other monomer is shown as a *gray ribbon*. A schematic view is used for those RdhA regions similar in structure to QueG, with similar color coding. Other regions (N-terminal and C-terminal extensions, larger insertions) are shown in *green ribbon* view. The bound cofactors for both structures are shown as atom color sticks with *spheres* for the Fe, S, and Co atoms.

**FIGURE 8.**
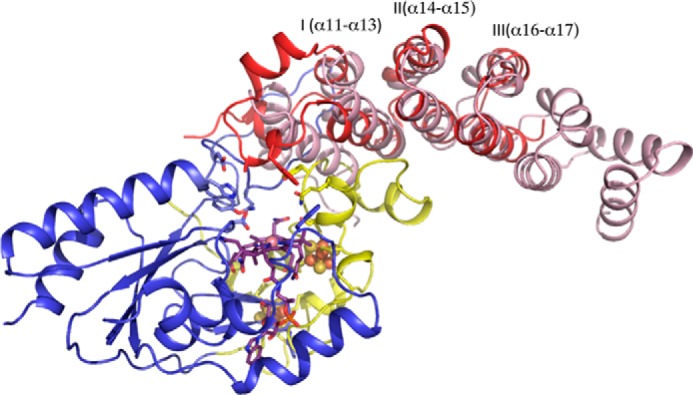
**Overlay of the QueG C-terminal domain with an artificially created tandem α-helical repeat protein (in *pink*).** Three distinct HEAT repeats can be discerned in QueG, the first containing an insert between the two HEAT α-helices that contains residues contributing to the active site (Ala-285 and Trp-288). The concave face of the QueG C-terminal domain (usually associated with protein:protein or protein:nucleic acids interactions in other tandem α-helical repeat proteins) packs against an extended loop region of the ferredoxin domain.

Indeed, despite the limited sequence homology with the reductive dehalogenases, the QueG cobalamin and [4Fe-4S] binding regions are remarkably similar to the corresponding reductive dehalogenase core region (Fig. 7). The tetrachloroethene reductase RdhA enzyme from *Sulfospirillum multivorans* (PDB code 4UQU; (14)) is most similar to *S. thermophilus* QueG, and can be aligned with an overall r.m.s.d. of 2.9 Å for 197 Cα, with a Z-score of 15.5 and a sequence identity of 17%. The C-terminal tandem α-helical repeat domain specific to QueG contains three HEAT repeats (31) and is most similar to an artificially created α-helical repeat protein (32); PDB code 3LTJ; r.m.s.d. 2.5 Å for 93 Cα with a Z-score of 9.8 and a sequence identity of 17%) (Fig. 8).

**FIGURE 9.**
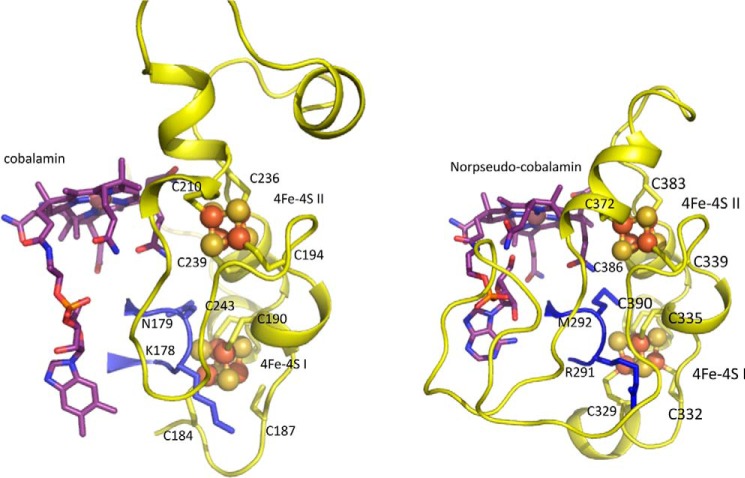
**Comparison between the QueG (*left panel*) and *S. multivorans* RdhA (*right panel*) ferredoxin modules, color coding as in Fig. 6.** Despite several insertions and deletions, the relative position of both [4Fe-4S] clusters is almost identical. In both enzymes, a conserved loop from the cobalamin binding domain provides part of the cluster I environment while the cobalamin cofactor itself shields one face of cluster II. For both enzymes, neither of the clusters is buried deep within the protein, suggesting interprotein electron transfer could occur to either cluster. In the case of QueG, significant disorder is observed for the Cys184-Cys187 connecting loop region.

While various insertions and deletions introduce substantial differences between both the QueG and the RdhA enzymes, the relative position of the [4Fe-4S] clusters with respect to the cobalamin is conserved (Fig. 9). The closest Fe-Fe distance between both [4Fe-4S] sulfur clusters is 9.5 Å in QueG, compared with 9.3 Å for the *S. multivorans* RdhA. The second iron sulfur cluster is in van der Waals contact with the cobalamin, and the closest Fe-to porphyrin distance for QueG is 6.3 Å compared with 5.2 Å for *S. multivorans* RdhA (Fig. 6). As observed for the reductive dehalogenases, both [4Fe-4S] clusters are located close to the protein surface, suggesting that in principle interprotein electron transfer is possible to either cluster.

In QueG, high levels of disorder can be observed for a loop region connecting two of the cysteine ligands (Cys-184 and Cys-187) to the first [4Fe-4S] cluster, suggesting the possibility of ligand heterogeneity for this particular cofactor. The extreme mobility of the loop including Cys-184, Gly-185, Asp-186, and Cys-187 accounts for the unusual g values and g strain exhibited by the spectrum of Fig. 5*D*, the geometries available to the cluster with two such mobile cysteine ligands being many and this being further enhanced by the variable interaction with the charge on Asp-186. These considerations effectively assign the EPR spectrum of Fig. 5*D* to the [4Fe-4S] cluster distant from the B12 cofactor (ligated by cysteines 184, 187, 190, and 243) while the sharper signal of Fig. 5*C* must arise from the [4Fe-4S] cluster close to the B12 cofactor (ligated by cysteines 194, 210, 236, and 239).

**FIGURE 10.**
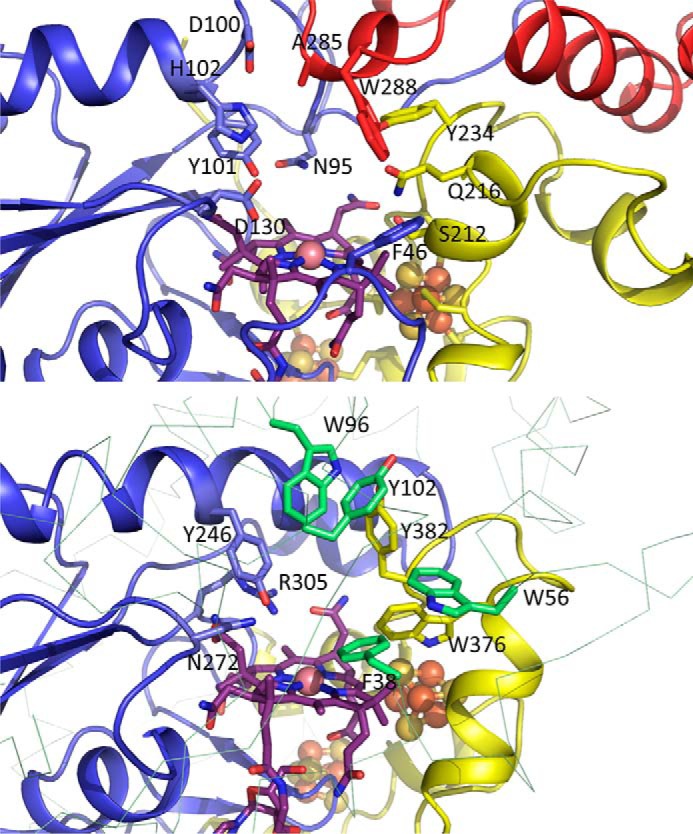
**Comparison between the QueG (*top panel*) and *S. multivorans* RdhA (*bottom panel*) active site regions, color coding as in Fig. 6.** Both proteins are shown in similar orientation viewed approximately perpendicular to the cobalamin corrin plane. With exception of the QueG Tyr-101/RdhA Tyr-246 and QueG D130/RdhA Asn-272 positions, there is little similarity between both active site architectures.

In contrast to the marked similarity of the QueG and RdhA redox chains, likely reflecting a common intraprotein electron transfer mechanism, substantial differences in the respective active site regions reflect the distinct nature of the substrate and the chemistry catalyzed by these enzymes (Fig. 10). In QueG, the active site contains residues contributed by the three distinct regions, many of which are strictly conserved in QueG homologs (Fig. 2). One side of the active site cavity is markedly more hydrophilic compared with the opposite face, and consists of the strictly conserved D100-Y101-H102 QueG motif in addition to the conserved D130. Previous mutagenesis studies with the *B. subtilis* QueG revealed mutation of each these residues to alanine significantly affected *in vivo* activity as well as the cobalamin spectroscopic properties (12). A putative salt bridge network is observed between D100-H102 and Asp-130, the latter residue pointing directly toward the cobalt fifth ligand region. Tyrosine 101 is located adjacent to D130 and similarly points directly to the cobalt water ligand, although neither is close enough to establish direct hydrogen bonds (both at ∼4.0 Å distance from the cobalt water ligand).

The opposite side of the cavity is markedly less hydrophilic in character, lined by Phe-46, Ser-212, Gln-216, Tyr-234, and Trp-288. None of these residues are strictly conserved in QueG homologs, suggesting the latter are unlikely to be directly involved in catalysis. A comparison with the reductive dehalogenase active site (Fig. 10) reveals RdhA enzymes contain an equivalent residue to the QueG Tyr-101, in the case of the *S. multivorans* RdhA Tyr-246. In the latter enzyme, Tyr-246 is in close contact to Asn-272 (which aligns with QueG Asp-130) and Arg-305. In RdhA, the conserved Tyr-246 to Arg-305 motif has been implicated in proton transfer to the organohalide substrate (13, 14). However, no equivalent residue to Arg-305 is present in QueG.

##### Modeling the QueG:Substrate Complex

**FIGURE 11.**
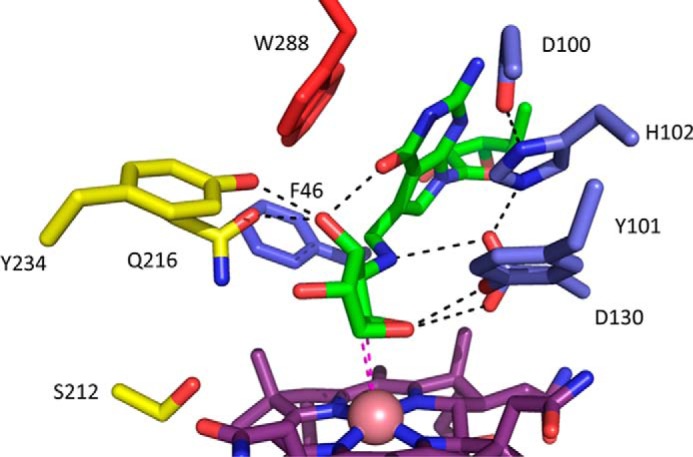
**Structure of the docked epoxyqueuosine:QueG complex.** Key active site residues are shown in atom color sticks, with carbons color coded as in Fig. 6. The docked substrate is shown with *green carbons*, and putative polar interactions made are shown by *dotted black lines*. Two *dotted magenta lines* indicate the close proximity between the epoxide carbons and the cobalamin cobalt ion.

The QueG oQ-tRNAsubstrate cannot be readily obtained, and we have used docking of epoxyqueuosine to QueG as an alternative means of probing the QueG-ligand interactions. Docking of the epoxyqueuosine ligand reveals a plausible model for the QueG-substrate complex, with the epoxypentanediol moiety directly above the cobalamin and the epoxide hydrogen bonding to both Tyr-101 and Asp-130 (Fig. 11). Further polar interactions are observed with the pentane diol groups and Gln-216 and Tyr-234 as well as a putative salt bridge between the epoxyQ amine group and Asp-130. In addition, the 7-deazapurine core forms a pi-pi stacking interaction with Trp-288. In the absence of further experimental data regarding QueG:epoxyQ-tRNA substrate interaction to guide modeling, we did not model the complete epoxyQ-tRNA. It is however attractive to speculate that the QueG specific C-terminal tandem α-helical repeat domain is involved in tRNA binding. Tandem α-helical repeats are frequently implicated in protein:protein or protein:nucleic acid contacts (33).

**FIGURE 12.**
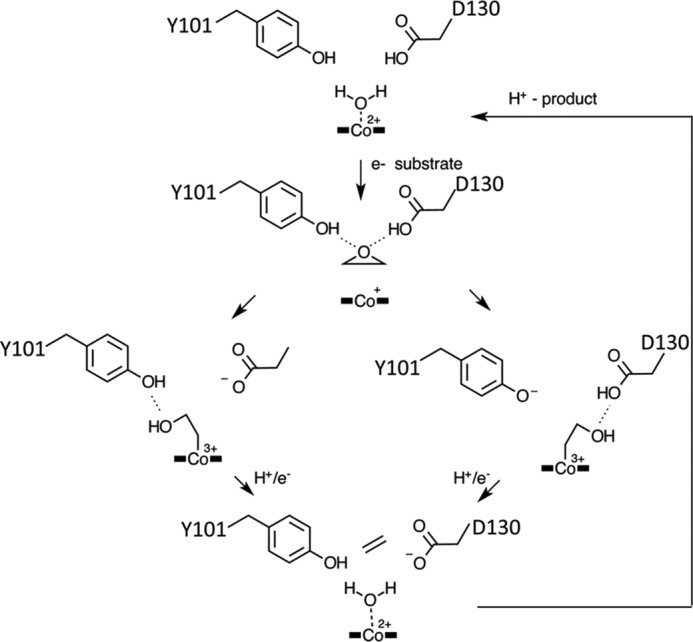
**Proposed mechanism for QueG epoxide reduction.** For clarity, only the epoxide group of the epoxyqueuosine-tRNA substrate is shown. We propose nucleophilic attack by Co(I) on one of the epoxide carbon atoms will result in one of two possible transient Co(III)-C substrate adducts. Rapid reduction by the [4Fe-4S] clusters to a corresponding Co(II)-alkyl adduct species is followed by heterolytic cleavage of this bond concomitant with water elimination leading to the cyclopentenediol product.

On the basis of our crystal structure data and the docked epoxyQ:QueG complex, we propose a mechanism for QueG involving direct nucleophilic attack by the reduced Cob(I)alamin (Fig. 12). The attack by Co(I) on either of the epoxide carbons could occur concomitant with proton transfer to the oxygen leaving group, either via conserved Asp-130 or Tyr-101 (both have been implicated in QueG enzyme activity (12)). We propose the resulting transient Co(III)-carbon linked enzyme:intermediate complex is rapidly reduced by the [4Fe-4S] clusters and leads to the queuosine product via heterolytic cleavage of the Co(II)-carbon bond in concert with a second protonation event (again via either Asp-130 or Tyr-101) to facilitate the elimination of water and formation of the cyclopentenediol group.

This proposed QueG mechanism is drastically different from the reductive dehalogenases, where formation of a transient carbon-cobalt adduct has been ruled out on active site steric considerations (13, 14). Instead, radical or halogen-cobalt chemistry based mechanisms have been proposed to underpin catalysis by reductive dehalogenases. At present, we cannot rule out alternative QueG mechanistic proposals that bear similarity to the latter, and the QueG structure provides a framework for future mechanistic studies aimed at establishing the exact nature of the various proton and electron transfer events during QueG catalysis.

## Discussion

Detailed structural and spectroscopic characterization of three distinct members from the Class III B12-dependent enzymes (11) is now available: two distantly related reductive dehalogenases (13, 14) in addition to the QueG epoxyqueusosine reductase (this report). Each of these contains a structurally conserved redox chain consisting of two closely spaced [4Fe-4S] clusters of which one is in direct van der Waals contact with the cobalamin corrin ring. In all cases, the cobalamin is bound in the pentacoordinate base-off form, facilitating formation of the Co(I) nucleophile. The human vitamin B12-processing enzyme exhibits a similar mode of cobalamin binding, but lacks the corresponding ferredoxin domain (30). In contrast to the conserved redox chain architecture of the cobalamin and [4Fe-4S]-dependent enzymes, the respective active sites are very distinct, reflecting the need to bind widely differing substrates (*i.e.* tetrachloroethene (14), dibromophenols (13), epoxyqueuosine-tRNA (12)). A single tyrosine residue positioned directly above the corrin is conserved in all three enzymes, and has been implicated in substrate proton transfer. However, the distinct protein environments surrounding the conserved tyrosine, combined with the widely differing chemical nature of the transformations catalyzed, likely suggest substantial diversity in mechanism for the Class III enzymes. In each case, the exact mechanism is yet to be fully elucidated, often focused on determining whether catalysis occurs *via* radical species. In addition to these mechanistic questions, little is known about the redox partners for any of the Class III enzymes, and this might well prove another source of diversity. Given the wide ranging nature of the established Class III enzymes, it is possible other yet uncharacterized B12- and [4Fe-4S] dependent enzyme families exist. However, a distinct cobalamin binding sequence motif is lacking for the class III enzymes, where only the bacterial ferredoxin two [4Fe-4S] cysteine ligation pattern is largely conserved (9). Therefore, discovery of further members of this class might have to await detailed characterization of a wide range of putative genes encoding C-terminal ferredoxin-like modules.

## Author Contributions

D. L. conceived and coordinated the study. K. A. P. P., K. F., H. S., and M. S. D. performed molecular biology and initial solution studies on a range of QueG enzymes that narrowed down plausible candidates for structural studies. K. A. P. P. and K. F. purified the *S. thermophilus* QueG; K. F. and S. E. J. R. carried out EPR and analyzed EPR spectroscopic data; K. A. P. P. and D. L. crystallized the enzyme and solved the crystal structure. B. B. and P. B. performed native MS experiments and analyzed the data. L. J. and S. H. performed docking calculation. K. A. P. P., S. E. J. R., and D. L. wrote the paper. All authors reviewed the results and approved the final version of the manuscript.

## Footnotes

4 The abbreviations used are:

tRNA: transfer RNA
Q: queuosine
oQ: epoxyqueuosine
RdhA: reductive dehalogenase
r.m.s.d.: root mean square deviation
CCP4: Collaborative Computational Project No. 4.
